# Supplementary material for: Raman Research on Bleomycin-Induced DNA Strand Breaks and Repair Processes in Living Cells
Source: Int J Mol Sci. 2022 Mar 24;23(7):3524. doi: 10.3390/ijms23073524 (PMC8998246; doi:10.3390/ijms23073524)
Supplement: Supplementary file 1 [file ijms-23-03524-s001.zip › ijms-1630008-supplementary.pdf]

Supplementary Material

Detection of bleomycin-induced DNA strand breaks and repair processes in living cells by Raman microscopy

Michał Czaja, Katarzyna Skirlińska-Nosek, Olga Adamczyk, Kamila Sofińska, Natalia Wilkosz, Zenon Rajfur, Marek Szymoński and Ewelina Lipiec*

M. Smoluchowski Institute of Physics, Jagiellonian University, 30-348 Kraków, Poland

michalandrzej.czaja@student.uj.edu.pl (M.C.); katarzyna.skirlinska@gmail.com (K.S.–N.); olga.adamczyk@doctoral.uj.edu.pl (O.A.); natalia.szydlowska@uj.edu.pl (N.W.); kamila.sofinska@uj.edu.pl (K.S.); sara.seweryn@doctoral.uj.edu.pl (S.S.); zenon.rajfur@uj.edu.pl (Z.R.)

*Correspondence: ewelina.lipiec@uj.edu.pl (E.L.)

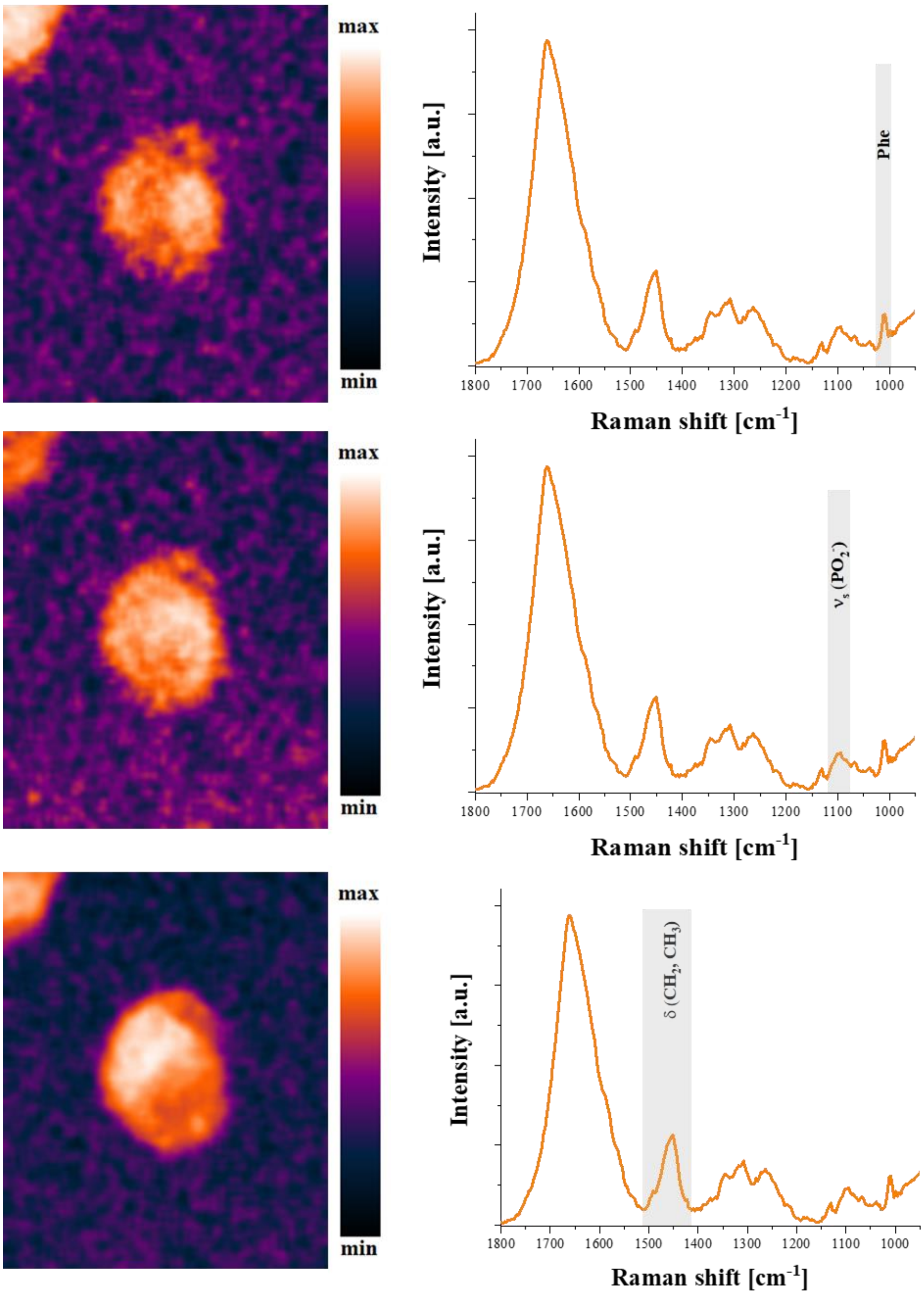

Figure S1. Raman maps presenting distribution of intensities of characteristic bands of specific biochemical compounds in control HeLa cell. At the top: distribution of proteins (Phe); center: nucleic acids and phospholipids $\nu_s(PO_2^-)$; bottom: lipids and proteins $\delta(CH_2, CH_3)$.

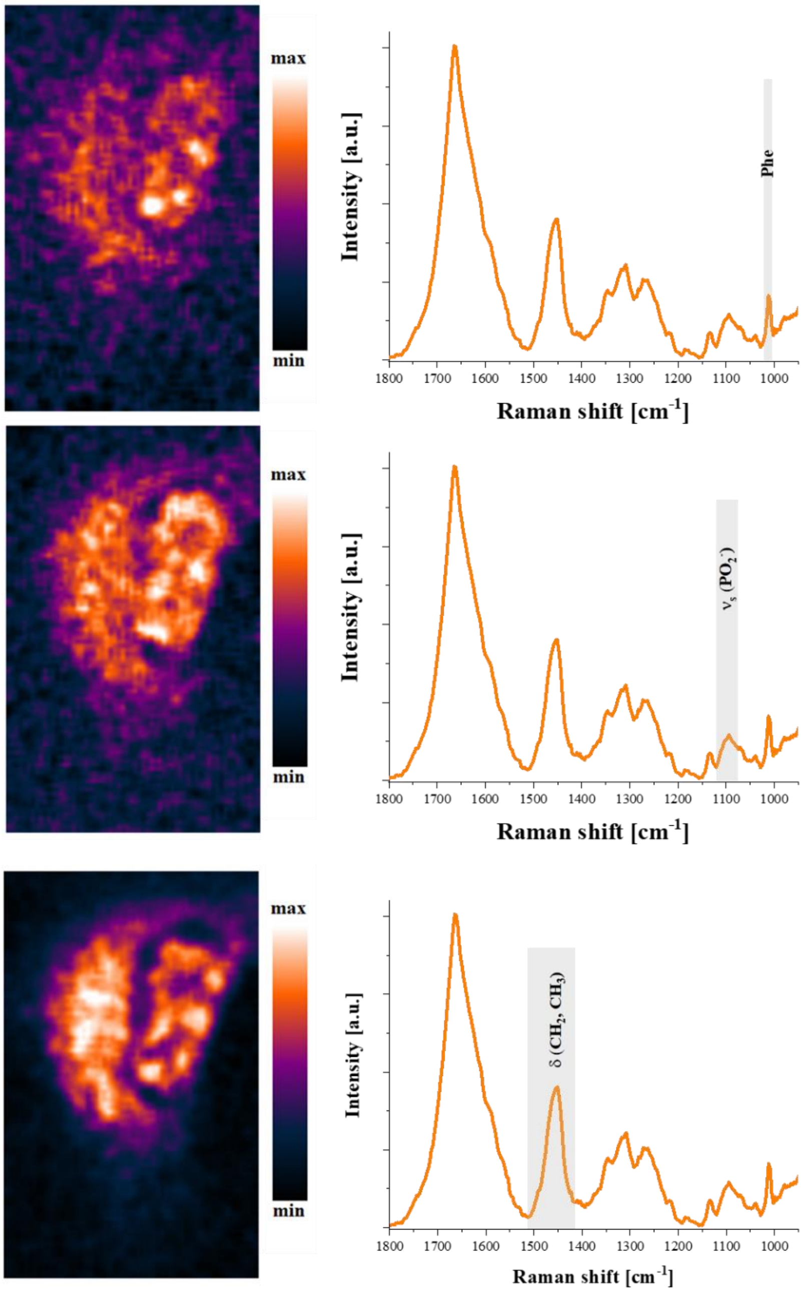

Figure S2. Raman maps presenting distribution of intensities of characteristic bands of specific biochemical compounds in HeLa cell incubated with 150 μM of bleomycin. At the top: distribution of proteins (Phe); center: nucleic acids and phospholipids $\nu_s(PO_2^-)$; bottom: lipids and proteins $\delta(CH_2, CH_3)$.

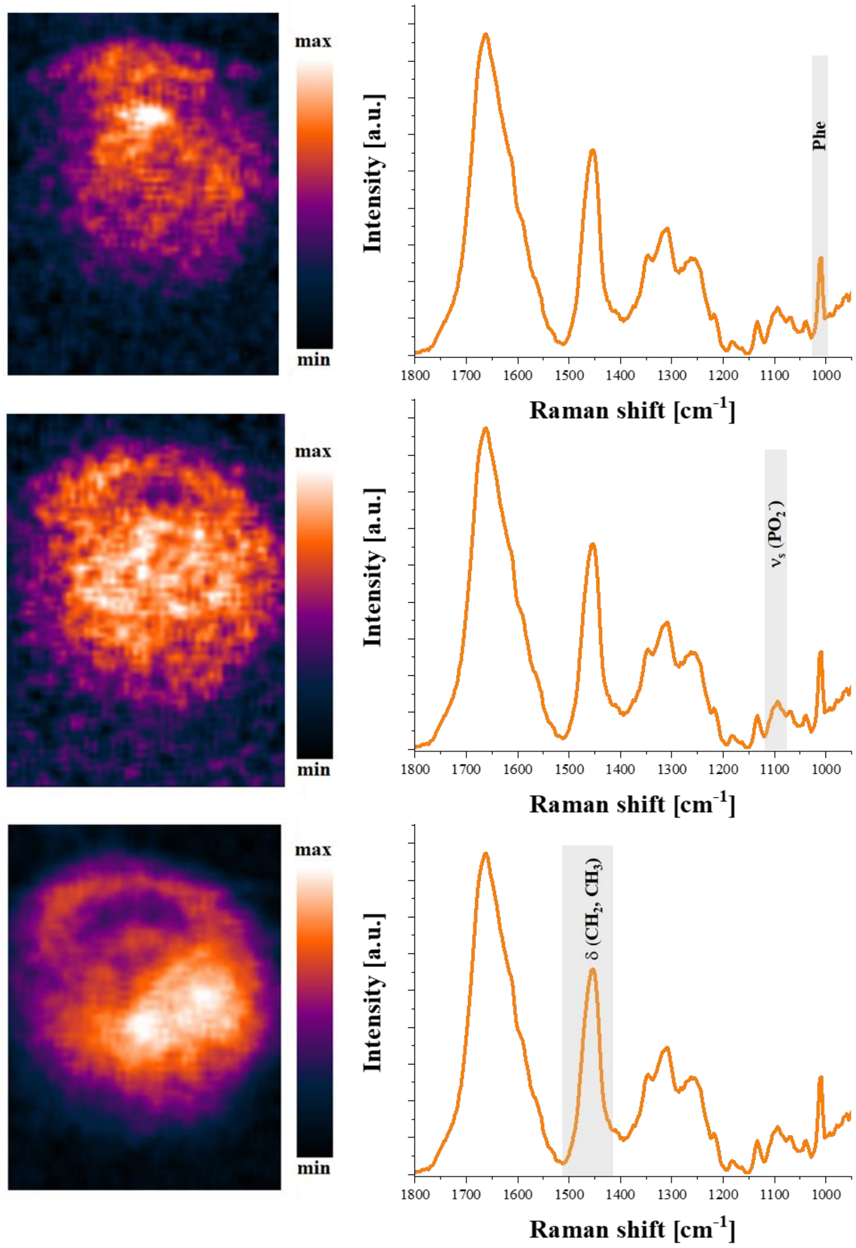

Figure S3. Raman maps presenting distribution of intensities of characteristic bands of specific biochemical compounds in HeLa cell incubated with 500 μM of bleomycin. At the top: distribution of proteins (Phe); center: nucleic acids and phospholipids $\nu_s(PO_2^-)$; bottom: lipids and proteins $\delta(CH_2, CH_3)$.

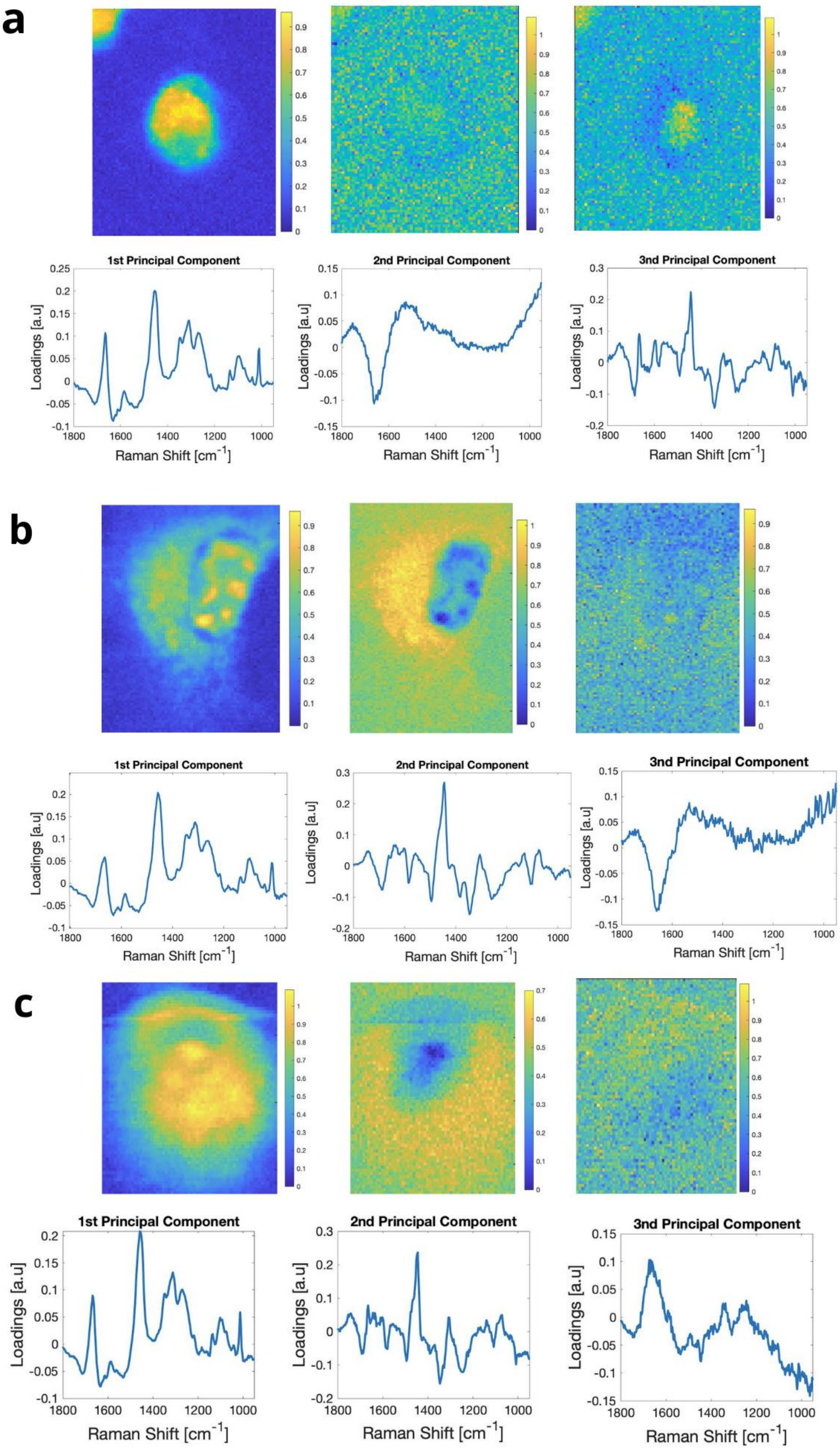

Figure S4. Distribution of PCs within Raman map of HeLa cells: (**a**) control, (**b**) incubated with 150 μM BLM, (**c**) incubated with 500 μM BLM. Loadings corresponding to three principal components are also presented.

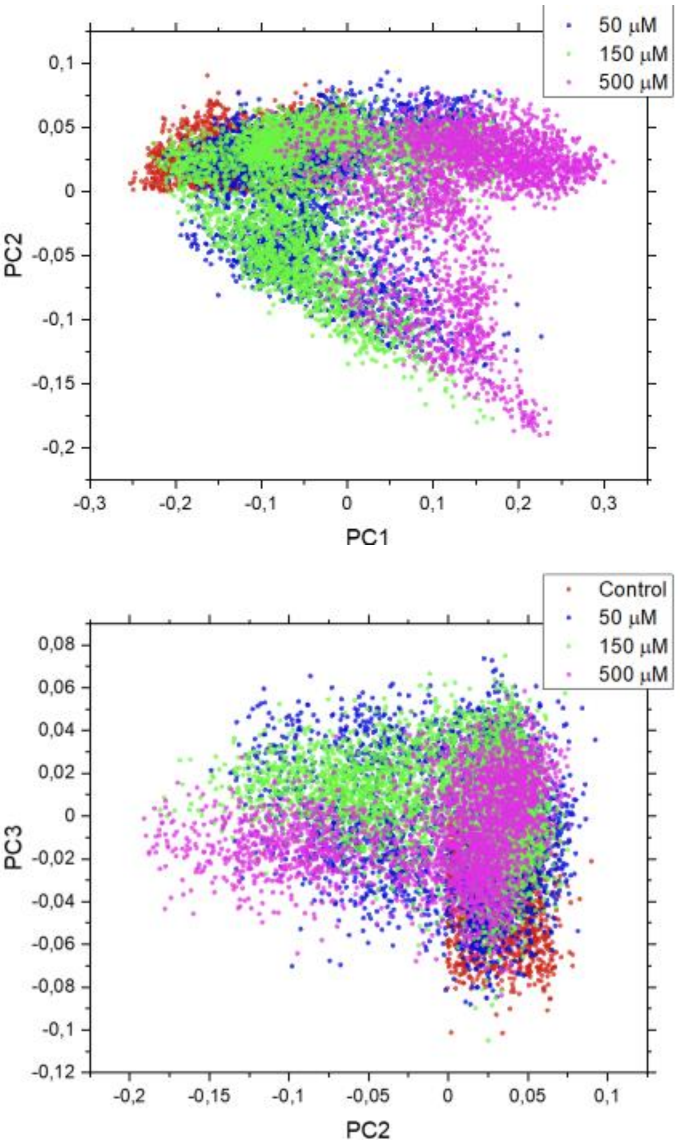

Figure S5. 2D PCA scores plots of Raman spectra of cellular nuclei of control HeLa cells and cells incubated with bleomycin (50 μM, 150 μM and 500 μM) for 24 h.

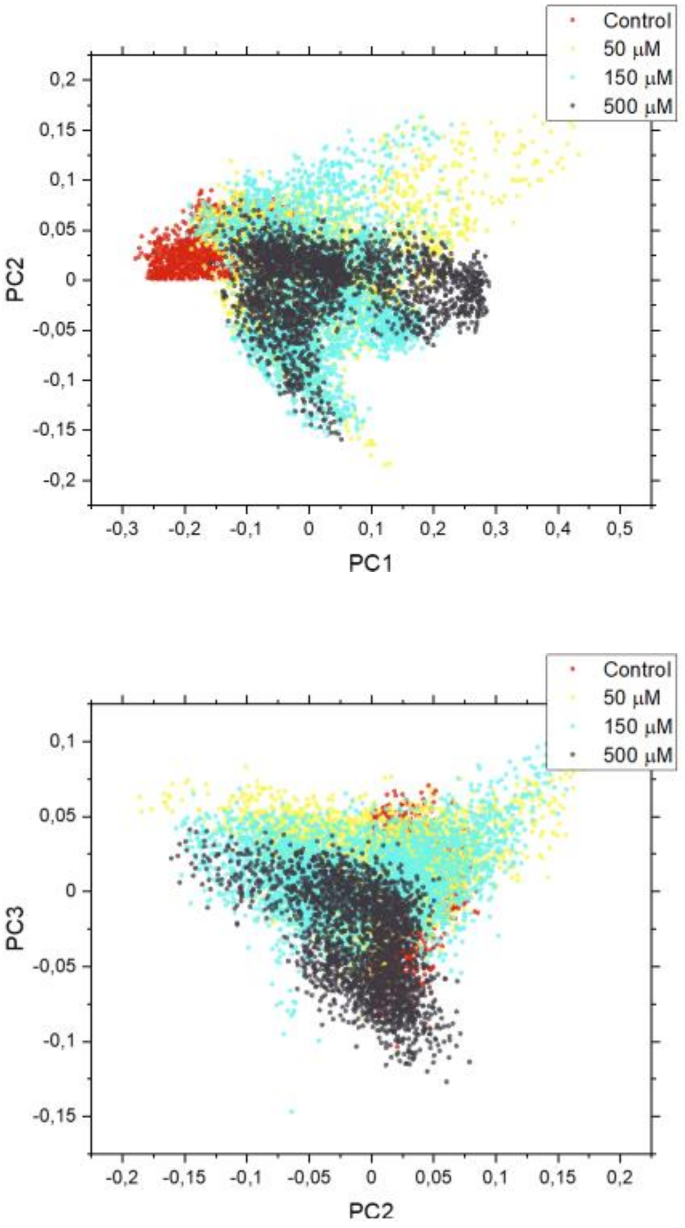

Figure S6. 2D PCA scores plots of Raman spectra of cellular nuclei of control HeLa cells and cells incubated with bleomycin (50 μM, 150 μM and 500 μM) for 48 h.

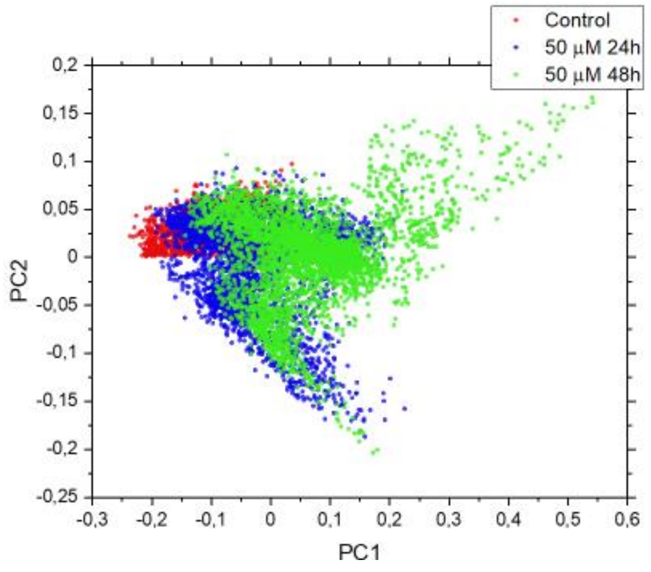

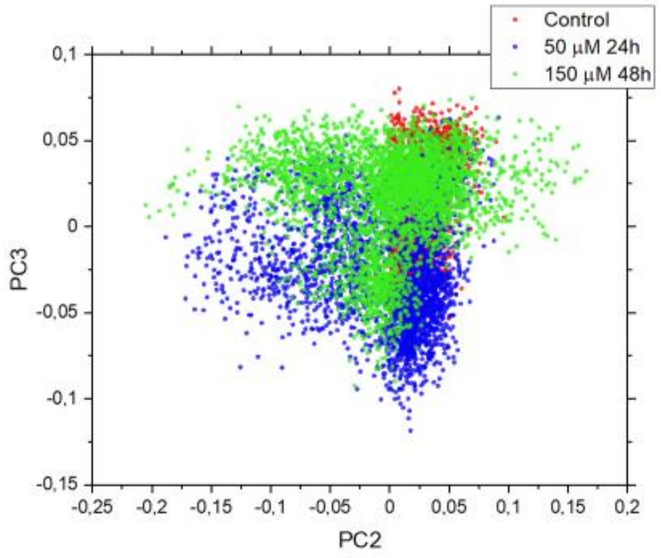

Figure S7. 2D PCA scores plots of Raman spectra acquired from nuclei of control cells and cells treated with 50 μM of BLM for 24 and 48 h.

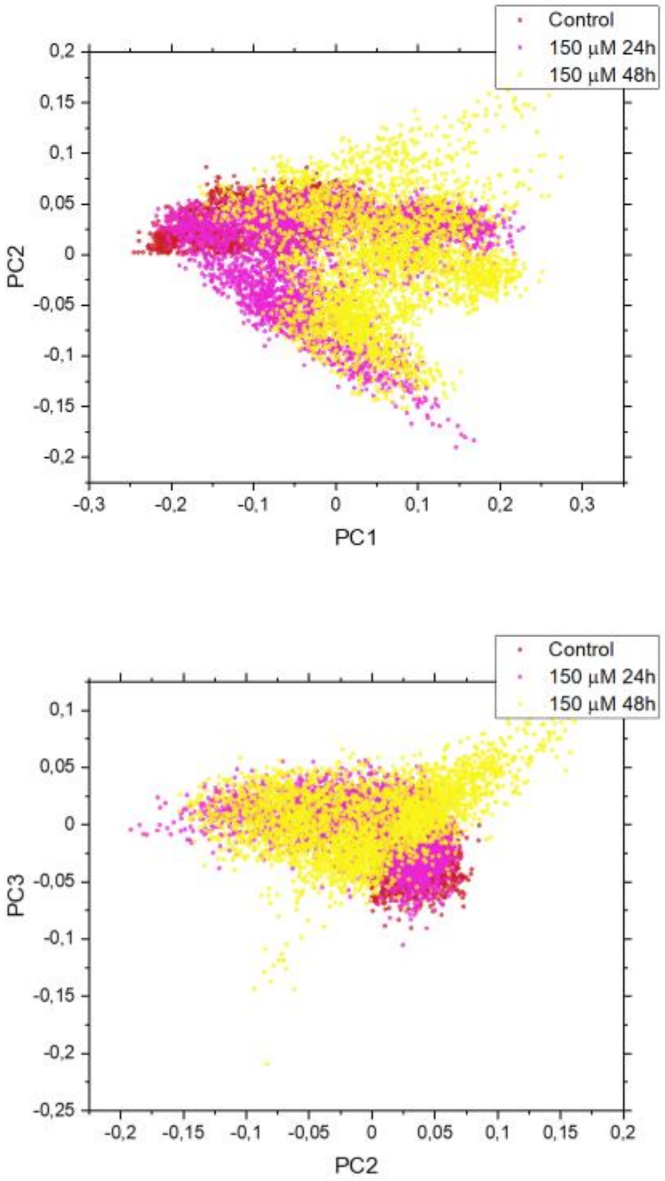

Figure S8. 2D PCA scores plots of Raman spectra acquired from nuclei of control cells and cells treated with 150 μM of BLM for 24 and 48 h.

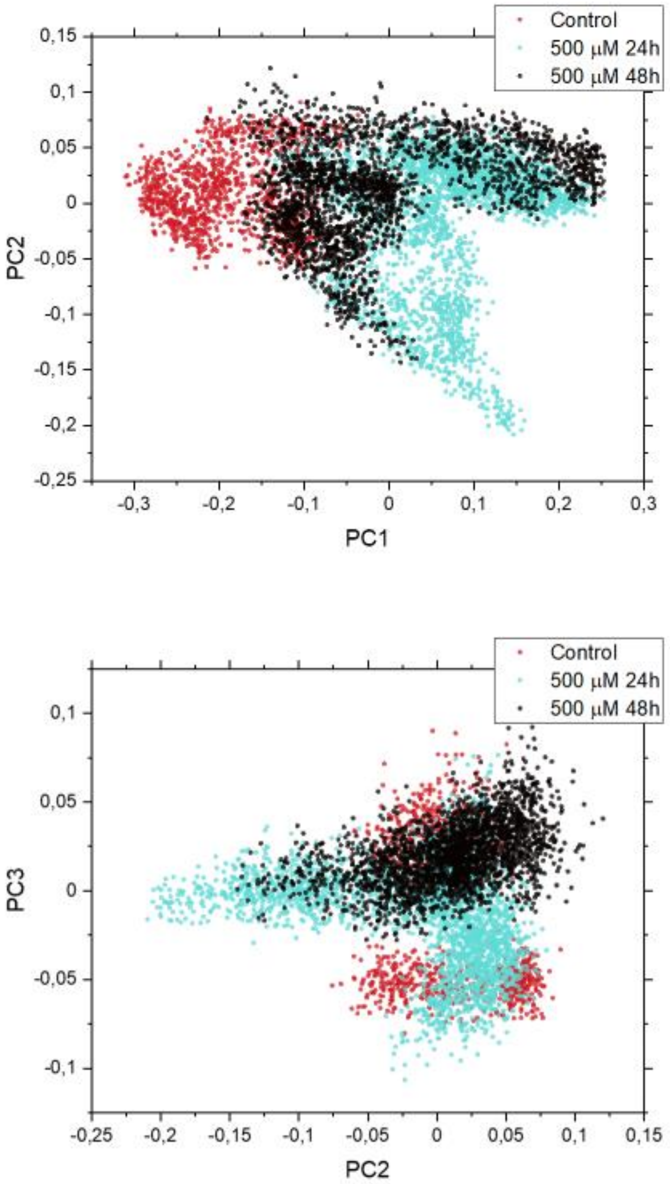

Figure S9. 2D PCA scores plots of Raman spectra acquired from nuclei of control cells and cells treated with 500 μM of BLM for 24 and 48 h.
